# Heterogeneous reduction of carbon dioxide by hydride-terminated silicon nanocrystals

**DOI:** 10.1038/ncomms12553

**Published:** 2016-08-23

**Authors:** Wei Sun, Chenxi Qian, Le He, Kulbir Kaur Ghuman, Annabelle P. Y. Wong, Jia Jia, Feysal M. Ali, Paul G. O’Brien, Laura M. Reyes, Thomas E. Wood, Amr S. Helmy, Charles A. Mims, Chandra Veer Singh, Geoffrey A. Ozin

**Affiliations:** 1 Department of Chemistry, Solar Fuels Research Cluster, University of Toronto, 80 St George Street, Toronto, Ontario, Canada M5S 3H6; 2 Institute of Functional Nano and Soft Materials (FUNSOM), Jiangsu Key Laboratory of Carbon-based Functional Materials and Devices, and Collaborative Innovation Center of Suzhou Nano Science and Technology, Soochow University, Suzhou, Jiangsu 215123, China; 3 Department of Materials Science and Engineering, University of Toronto, 184 College Street, Suite 140, Toronto, Ontario, Canada M5S 3E4; 4 Department of Chemical Engineering and Applied Chemistry, Solar Fuels Research Cluster, University of Toronto, 200 College Street, Suite 103, Toronto, Ontario, Canada M5S 3E4; 5 The Edward S. Rogers Sr Department of Electrical and Computer Engineering, University of Toronto, 10 King’s College Road, Toronto, Ontario, Canada M5S 3G4; 6 The Department of Mechanical and Industrial Engineering, University of Toronto, 5 King’s College Road, Toronto, Ontario, Canada M5S 3G8

## Abstract

Silicon constitutes 28% of the earth’s mass. Its high abundance, lack of toxicity and low cost coupled with its electrical and optical properties, make silicon unique among the semiconductors for converting sunlight into electricity. In the quest for semiconductors that can make chemicals and fuels from sunlight and carbon dioxide, unfortunately the best performers are invariably made from rare and expensive elements. Here we report the observation that hydride-terminated silicon nanocrystals with average diameter 3.5 nm, denoted ncSi:H, can function as a single component heterogeneous reducing agent for converting gaseous carbon dioxide selectively to carbon monoxide, at a rate of hundreds of μmol h^−1^ g^−1^. The large surface area, broadband visible to near infrared light harvesting and reducing power of SiH surface sites of ncSi:H, together play key roles in this conversion. Making use of the reducing power of nanostructured hydrides towards gaseous carbon dioxide is a conceptually distinct and commercially interesting strategy for making fuels directly from sunlight.

## Abstract (web summary)

Elemental silicon is widely studied for photovoltaic applications. Here, the authors report that hydride-terminated silicon nanocrystals can also function as single component heterogeneous reducing agent for converting gaseous carbon dioxide selectively to carbon monoxide.

## Graphical abstract

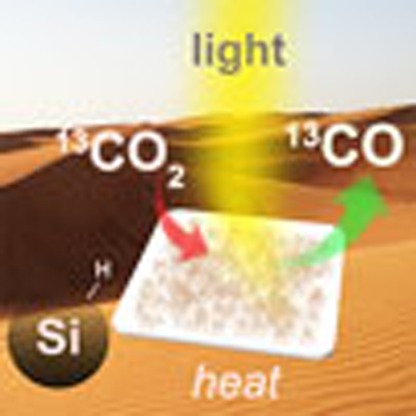

Owing to its unique electrical, optical and thermal properties as well as its high earth abundance, silicon materials have found pervasive applications in energy conversion and storage. Silicon nanostructures with large surface areas and widely tunable chemical and physical properties are of special interest in diverse areas, including solar cells^1^,^2^,^3^, lithium-ion batteries^4^,^5^,^6^,^7^, thermoelectrics^8^,^9^ and photocatalysis^10^.

Recently, the field of solar fuels has emerged that aims to harvest, convert and store solar energy in the form of chemical energy^11^. Owing to silicon’s low cost and lack of toxicity, and small electronic band gap for near-infrared to visible light absorption, it is scientifically and technologically interesting to introduce silicon nanostructures into different types of solar-to-chemical energy platforms. Silicon nanowires, for example, have been employed as electrodes for photoelectrochemical generation of H_2_ from H_2_O, photodegradation of dyes, and as anodes in lithium ion batteries^4^,^6^,^12^,^13^,^14^. Porous silicon and silicon nanocrystals are showing promise for bio-medical and optoelectronic applications^15^,^16^.

In the context of CO_2_ reduction, it is noteworthy that organo silyl hydrides are well known for their ability to homogeneously reduce CO_2_. In stark contrast, hydride functionalized silicon nanocrystals have not previously been imagined as a reagent for the heterogeneous gas-phase reduction of CO_2_. To amplify on the former, the first step in the solution phase hydrosilation of SiH bonds in molecular silyl hydrides with CO_2_ has been reported to involve the formation of a formoxysilane SiOCHO group containing a SiO bond^17^. This insertion reaction is usually enabled using a transition metal or main group homogenous co-catalyst under high temperature and/or pressure conditions. In the case of molecular silyl dihydrides, hydrosilation of CO_2_ has been shown to form di-formoxysilane Si(OCHO)_2_ groups^18^. These formoxysilanes as well as silylacetal groups have been implicated in the reduction of CO_2_ to CH_3_OH and CH_4_ (refs 17, 19, 20, 21, 22). There was also a report that by using aqueous Na_2_CO_3_ in the presence of silicon quantum dots, both HCHO and HCO_2_H were detected using the Nash reagent^23^.

Herein we document the ability of surface hydride functionalized silicon nanocrystals, denoted ncSi:H, to selectively reduce gaseous CO_2_ to CO using the heat and light from the sun. Compared with this gas-phase heterogeneous reduction of CO_2_ the aforementioned liquid-phase homogenous hydrogenation of CO_2_ has several disadvantages that include: (i) solubility, diffusion and temperature limitations of CO_2_ in the liquid-phase, (ii) requirement of a catalyst, (iii) recovery and regeneration of catalysts from the liquid-phase and (iv) the scalability of the process. A further advantage of ncSi:H is its ability to harvest light across the near-infrared to visible wavelength range which provides opportunities for photothermal reduction of CO_2_ using both the heat and light from the sun. If the reducing SiH surface of ncSi:H could be maintained under reaction conditions the reduction of CO_2_ could potentially be made catalytic.

## Results

### Synthesis and characterization of hydride-terminated ncSi

**Figure 1:**
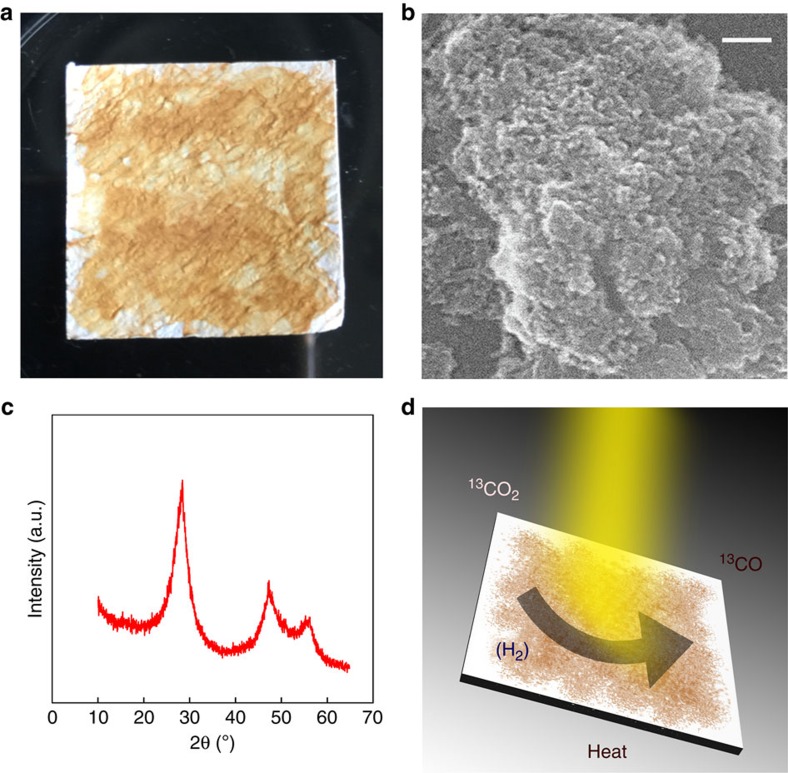
Characterization of hydride-terminated ncSi. (**a**) As-synthesized powder comprised of ncSi:H deposited on a borosilicate glass fibre filter support. (**b**) SEM image of a film sample made of dried ncSi:H from a pentane dispersion. Scale bar, 100 nm. (**c**) Powder X-ray diffraction pattern of ncSi:H, diffraction the main reflections peaks for nanocrystalline Si. (**d**) Schematic illustration of the reduction of CO_2_ to CO by ncSi:H.

Hydride-terminated silicon nanocrystals, denoted ncSi:H, were obtained through a two-step synthesis reported before^24^. The source of ncSi:H is silicon monoxide SiO, a low-cost commodity material available in kilogram quantities. Thermal treatment of SiO in a 5% H_2_/Ar environment causes a redox disproportionation reaction in which the formally Si(II) in SiO is simultaneously reduced to Si(0) and oxidized to Si(IV). The so-formed Si(0) undergoes nucleation and growth to form ncSi in a SiO_2_ matrix^25^. The size of the produced ncSi is within the range of 2–7 nm (ref. 24). Subsequent extraction of the ncSi from the SiO_2_ surrounding matrix is accomplished using aqueous HF, and the product is a brown powder comprised of ncSi:H (Fig. 1a). Owing to their small sizes, a notably large surface area of 368 m^2^ g^−1^ was determined for ncSi:H by nitrogen gas adsorption (Supplementary Fig. 1). Scanning electron microscopy (SEM) investigations of the ncSi:H samples show they consist of aggregates of nanocrystals with textural nanoporosity (Fig. 1b), which is consistent with the large surface area measured. If we regard such textural interstices as pores, the mode pore size is 3.5 nm and the pore volume is 0.381 cc g^−1^, determined from the nitrogen gas adsorption experiment (Supplementary Fig. 1). In Fig. 1c the powder X-ray diffraction pattern of a typical ncSi:H sample is depicted. All diffraction peaks can be assigned to silicon with no obvious ones, amorphous or crystalline, from SiO or SiO_2_ being detected. The surface of the obtained ncSi can be seen from Fourier transform infrared spectroscopy (FTIR) to contain plenty of Si:H bonds, providing the necessary capacity for reducing CO_2_ into CO (Fig. 1d). The ultraviolet–vis diffuse reflectance spectra of the brown ncSi:H sample shows a strong broad-band optical absorption increasing in absorptivity as it traverses from the near infrared to ultraviolet wavelength range arising from a convolution of quantum size effects in the ncSi:H size distribution (Supplementary Fig. 2), rendering ncSi:H a potentially effective photothermal CO_2_ reducing agent.

### CO production from CO_2_ in the absence and presence of H_2_

**Figure 2:**
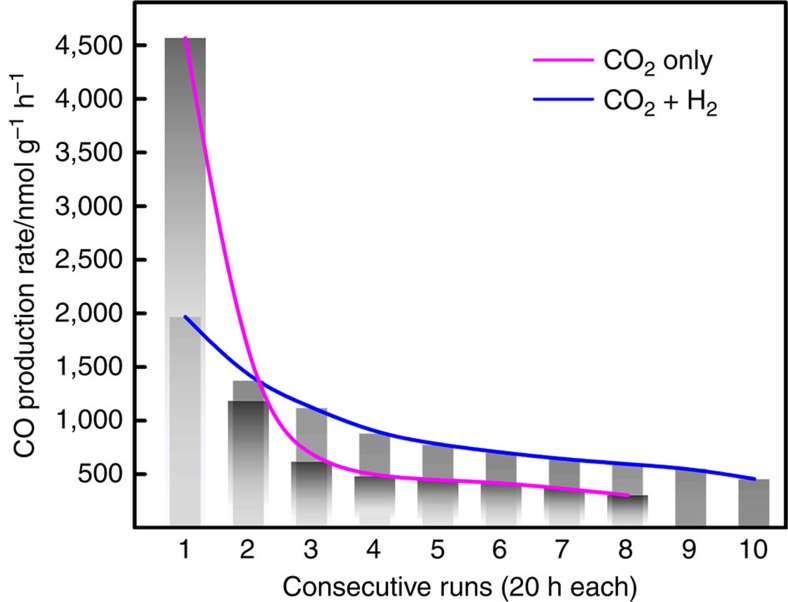
Light-driven CO_2_ reduction. CO production rates (pure ^13^CO_2_ in the batch reactor at 150 °C) for eight cycles, and CO production rates (^13^CO_2_ and H_2_ with 1:1 ratio in the batch reactor at 150 °C) for 10 cycles, both under light (one sun) with the same total pressure of 27 p.s.i.

The reactivity of these ncSi:H samples towards CO_2_ was first studied in a batch reactor irradiated with a metal halide lamp, in the absence and presence of H_2_ at 150 °C, for multiple cycles (Fig. 2). To ensure the products of the reactions did not originate from adventitious carbon residues in the ncSi:H samples, isotope labelled ^13^CO_2_ was used to authenticate the origin of the reduction reaction. In the absence of H_2_, we observed a notable initial CO production rate as high as 4.5 μmol h^−1^ g^−1^ for an incident solar intensity of 1 sun at 150 °C. The rate decreased in the following cycles but the sample was still active over 160 h (Fig. 2). It is important to note that ^13^CO was found as the dominant product and no other ^13^C-containing compounds were detected (except for unreacted ^13^CO_2_). The results of the batch experiments unequivocally demonstrate that ncSi:H itself can heterogeneously reduce CO_2_:




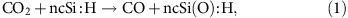




where ncSi(O):H stands for surface oxidized ncSi:H.

With both H_2_ and CO_2_ in the reactor, the initial CO production rate is about half of that for the case with the presence of only CO_2_, as expected because the partial pressure of CO_2_ is cut by half. This further confirms that CO_2_ was indeed a reactant. Notably, the rate of subsequent runs decreases much more slowly (Fig. 2), showing different kinetics, which suggests H_2_ likely gets involved in the CO_2_ reduction process. Unlike the case of only CO_2_ present in the reactor, where the CO production rate dropped significantly even at the second run, we instead observed an increased CO production rate when we introduced H_2_ to the reactor (Supplementary Fig. 3). Although the following tests showed the rate was still gradually decreasing, the presence of H_2_ seemed to aid in retaining more active sites, for example inhibiting the reaction between Si–H and adsorbed/product H_2_O to yield H_2_ (ref. 14).




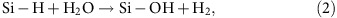




according to the simple Le Chatelier’s principle, and similarly inhibiting the hydride loss to released H_2_ on heating^26^:




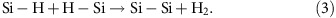




**Figure 3:**
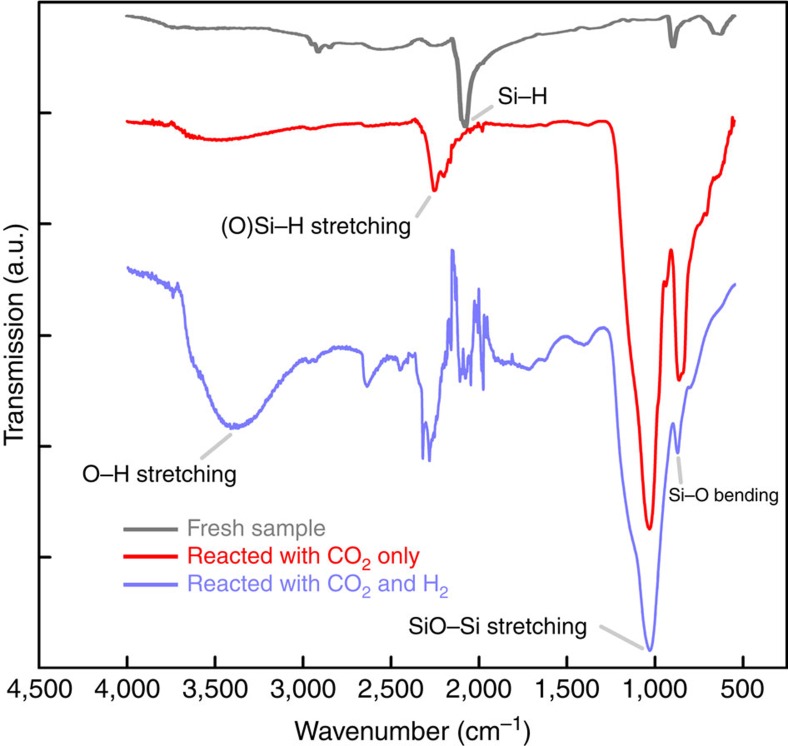
FTIR study of surface properties. FTIR spectra of fresh ncSi:H powder before the reaction, after the reaction with ^13^CO_2_ only for over 160 h, and after the reaction with both ^13^CO_2_ and H_2_ for over 200 h.

As discussed later, the FTIR spectrum of ncSi:H after reaction with both H_2_ and CO_2_ also exhibits less surface Si–O–Si but more Si–OH, compared with that of ncSi:H reacted with only CO_2_ (Fig. 3). Importantly, the ultraviolet–vis diffuse reflectance spectrum is less blue-shifted, again indicating lesser amounts of surface oxidation (Supplementary Fig. 2).

To further understand the reaction mechanism, all ncSi:H samples were studied by FTIR spectroscopy before and after testing, to gain an insight into the surface chemistry responsible for the products formed from the reaction of CO_2_ and CO_2_/H_2_ with ncSi:H, respectively. FTIR studies of the ncSi:H samples subjected to the testing conditions described above, before and after exposure to CO_2_ or CO_2_/H_2_ reactants for many cycles are shown in Fig. 3. The FTIR spectra indicate that before the reaction, the characteristic Si–H stretching mode is dominant on the ncSi:H surface, with a little amount of residual CH_x_ species from pentane extraction observed at ∼2,900 cm^−1^. After reaction with only CO_2_ for a significant number of cycles, accompanied by the aforementioned decrease of the CO production rate, the surface of ncSi:H was oxidized and Si–O–Si and Si–OH species were formed, which resembles the situation for oxidized Si nanowire surfaces^27^. Interestingly, there was still considerable amounts of OSi–H species remaining on the surface seen at around 2,250 cm^−1^. Compared with the original SiSi–H, the hydride peak shifted from around 2,100 cm^−1^ to larger wavenumbers, which is diagnostic of some surface oxidation^28^. These results suggest that only the non-oxidized related surface Si–H sites are responsible for the reduction of CO_2_ to CO, and the reaction is most likely stoichiometric through O transfer from CO_2_ to the surface of ncSi:H. This is supported by the result of a control test, in which a ncSi:H film was oxidized in air and daylight conditions for weeks, and showed no detectable production of CO. In contrast, in the presence of H_2_, a higher proportion of surface Si–OH groups are formed after reacting for a similar amount of time, which again implies H_2_ may play a role in the CO_2_ reduction process. Nevertheless, with our test condition the presence of H_2_ could not reinstate the non-oxidized Si–H surface, thus the reaction was still considered not catalytic. Note that the residual organics are being removed during reaction (Fig. 3) and are not the source of reducing ^13^CO_2_ to ^13^CO. This is further confirmed by a control test where we intentionally grafted a decyl group onto ncSi via hydrosilylation. The ^13^CO did not increase but rather dropped significantly proving that the Si–H is the active site.

**Figure 4:**
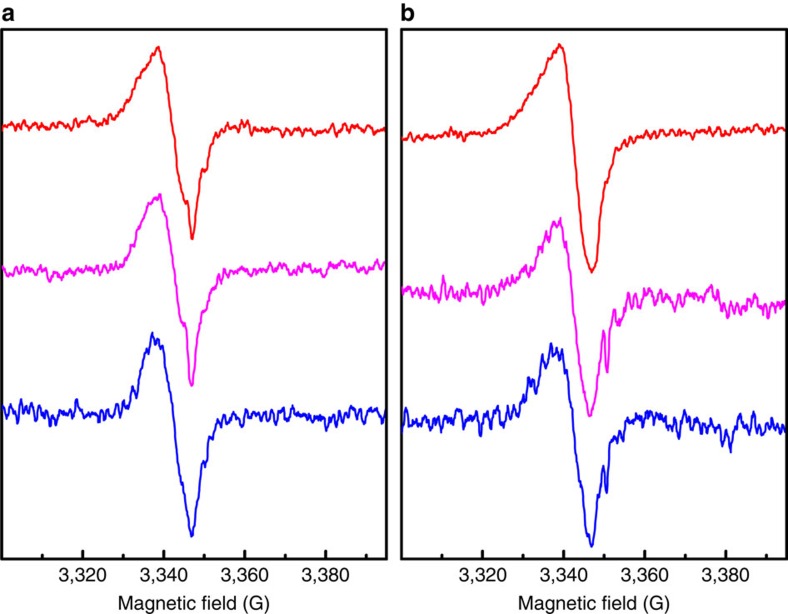
EPR studies. EPR spectra of ncSi:H samples (**a**) in N_2_ (red, at room temperature; pink, at 170 °C in dark; blue, at 170 °C with 0.5 sun); microwave frequency, 9.393 GHz, (**b**) in CO_2_ (red, at room temperature; pink, at 170 °C in dark for over half an hour; blue, at 170 °C with 0.5 sun for another half an hour); microwave frequency, 9.393 GHz.

The surface oxidation was further confirmed by probing the dangling bonds on ncSi:H by electron paramagnetic resonance (EPR). We observed split peaks for the sample sealed in CO_2_ when the temperature was increased to 170 °C both in dark and light (Fig. 4b). The small peaks at 3,351 G and at 3,332 G were hardly seen for the control sample under N_2_ (Fig. 4a). While the main signals (in all six spectra) are attributed to silicon dangling bonds in a disordered environment (with g∼2.0081)^28^,^29^, the new feature that emerged in the presence of CO_2_ should originate from the generation of the interface between the ncSi core and the surface oxidation shell. This new feature is similar to the well-known P_*b*_ centre in bulk Si/SiO_2_ interfaces^28^,^30^,^31^, with an observed *g* value *g*_∥_=2.0027.

### Effects of light and temperature

**Figure 5:**
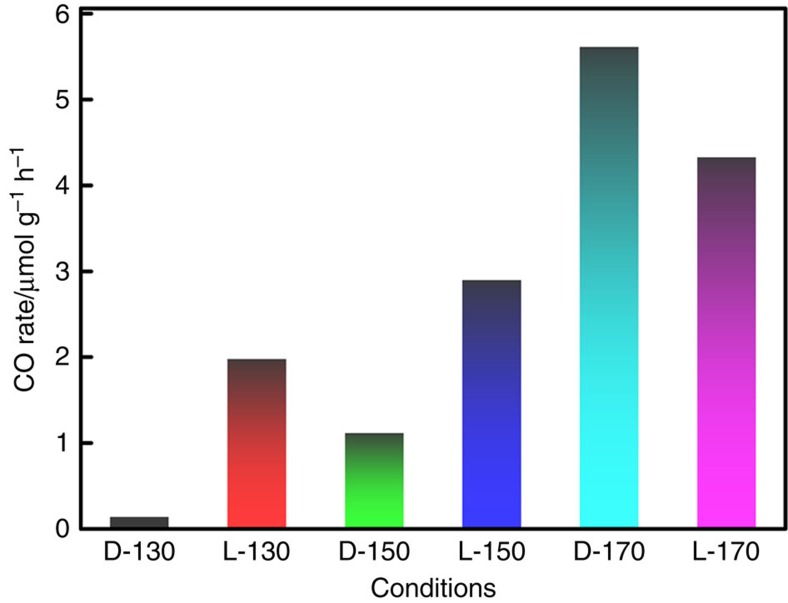
Temperature effect. CO production rates of three ncSi:H film samples tested at different heating temperatures (130 °C, 150 °C, and 170 °C), first in the dark (D) and then in the light (L).

Our standard test condition includes irradiation with light, but the same reaction to reduce CO_2_ to CO can also be enabled thermally. In the absence of illumination, detectable amounts of CO can be observed after reaction over 20 h at around 130 °C. The production rate quickly increased to 1 μmol h^−1^ g^−1^ at 150 °C and further exceeded 5 μmol h^−1^ g^−1^ at 170 °C (Fig. 5). In most cases, the samples show higher CO production rates in the light at the same heating temperature except in the case of 170 °C when the sample shows slightly higher CO production rate in the dark. The reason is that the studies on the temperature and light effects shown in Fig. 5 were conducted using the same sample for each temperature, following the sequence of first in dark then in light, being consistent throughout the whole experiment. Therefore there might be less reactive sites for the light test after the dark test was done, showing the same decreasing trend for the consecutive runs shown in Fig. 2. Instead, when a fresh ncSi:H sample was directly tested at 170 °C in light, the CO production rate for the first run reaches up to 17 μmol h^−1^ g^−1^, roughly three times of that in the dark at the same temperature.

**Figure 6:**
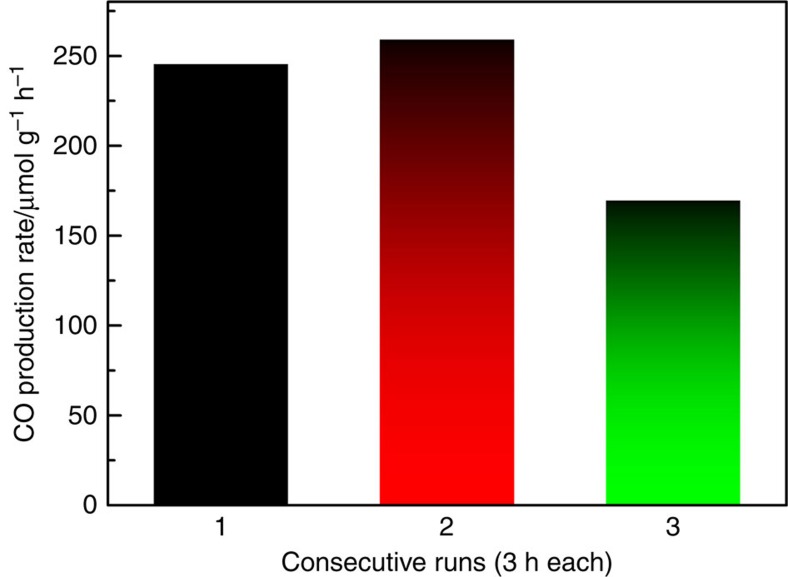
Light source effect. Enhanced CO production rates of a ncSi:H sample illuminated with ∼15 suns.

We do not observe new products other than ^13^CO and the EPR results show very similar patterns regardless of the irradiation with light (Fig. 4). Most importantly with only light irradiation at the intensity of one sun but without heating we did not obtain observable amounts of ^13^CO. Thus we attribute the enhanced conversion rate to the photothermal effect of ncSi:H. In other words, the local temperature at the ncSi:H surface was higher with light irradiation than in the dark under similar thermal heating conditions, which is responsible for the observed light enhancement effect. Significant photothermal effects of Si nanostructure have been well documented in recent years. For example, the CO_2_ reduction rate of ruthenium nanoparticles was found to be greatly enhanced by the photothermal effect of black Si nanowires^32^. Porous Si nanoparticles have been used for photothermal therapy^33^. For Si nanocrystals, laser light could induce intense local heating^34^, and the photothermal effect of ncSi increases with irradiation energy, consistent with a combination of thermalization of hot carriers under irradiation greater in energy than the bandgap (ultraviolet/visible light) and defect-mediated heating processes (induced by both ultraviolet/visible light and infrared light)^35^. A preliminary Raman study also shows that laser light could significantly heat up Si nanocrystals embedded in SiO_x_ matrix (Supplementary Fig. 4). As shown in Fig. 5, the enhancement effect is more pronounced at 130 °C owing to the largest local temperature difference at the ncSi:H surface between dark and light conditions. Intuitively, the conversion rate could be further improved with more incident photons reaching the ncSi:H surface. To further confirm the light enhancement mechanism, we also performed the reaction in CO_2_ and H_2_ at 150 °C but with the irradiation of significantly higher intensity light from a Xe lamp (∼15 suns). Amazingly, a 100 times boost of the rate to ∼250 μmol h^−1^ g^−1^ was observed, which is comparable to the best reported CO_2_ reduction catalysts (Fig. 6). It is notable that it is convenient to achieve 15 suns of light under daily sunlight with the use of a commercial solar concentrator. When illuminated with more concentrated light with the intensity of 20 suns, significant amounts of CO can be produced without providing external heating (Supplementary Fig. 5), but the ^13^CO rate was still decreasing over consecutive runs even in the presence of H_2_ (Supplementary Table 1), consistent with the decreasing trend observed under the other reaction conditions (Supplementary Tables 1–3). While the exact role of H_2_ in the reduction process needs further study, it is believed that the presence of H_2_ used in these experiments is beneficial for maintaining the reductive atmosphere that minimizes the undesired surface oxidation and dehydrogenation of ncSi:H^36^.

### Stability of hydride-terminated ncSi in air

**Figure 7:**
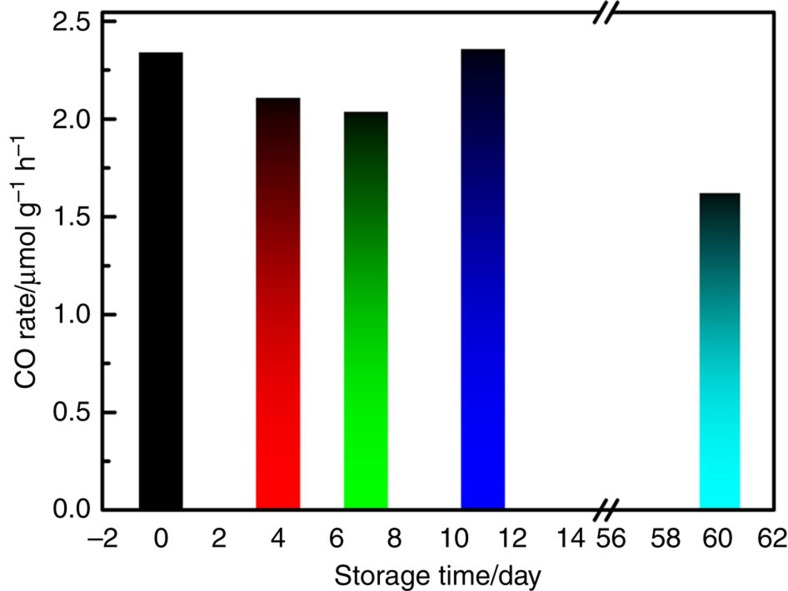
Stability of hydride-terminated ncSi during storage. CO production rate of ncSi:H film samples after different time spans of storage, which demonstrates the stability of the ncSi:H sample in air without the exposure to light.

As discussed above, the reductive surface of ncSi:H is crucial for converting CO_2_ into CO. A common concern would be the stability of ncSi:H against oxidation during storage. One may expect that only freshly prepared samples are active and must be handled under humidity and oxygen free conditions, which may not be convenient for scale-up for industrial applications. Contrary to common belief, our ncSi:H powder is surprisingly stable if stored in air with proper care. For example, throughout an 11-day storage in a dark cabinet, the CO production rate was still as high as the rate shown by the fresh sample (Fig. 7). Even after an incredibly long time of two months, the rate only dropped a little to 1.62 μmol h^−1^ g^−1^. Our FTIR results also implied very little oxidation with much of the Si–H preserved under such conditions (Supplementary Fig. 6). However, these nanocrystals quickly lost their reducing power stored under similar conditions but exposed to ambient room light. Apparently, the oxidation of the ncSi:H surface can be greatly accelerated even with ambient light^37^. Therefore, the key to maintain the reductive surface of ncSi:H during the storage in air is away from light.

### DFT simulations

**Figure 8:**
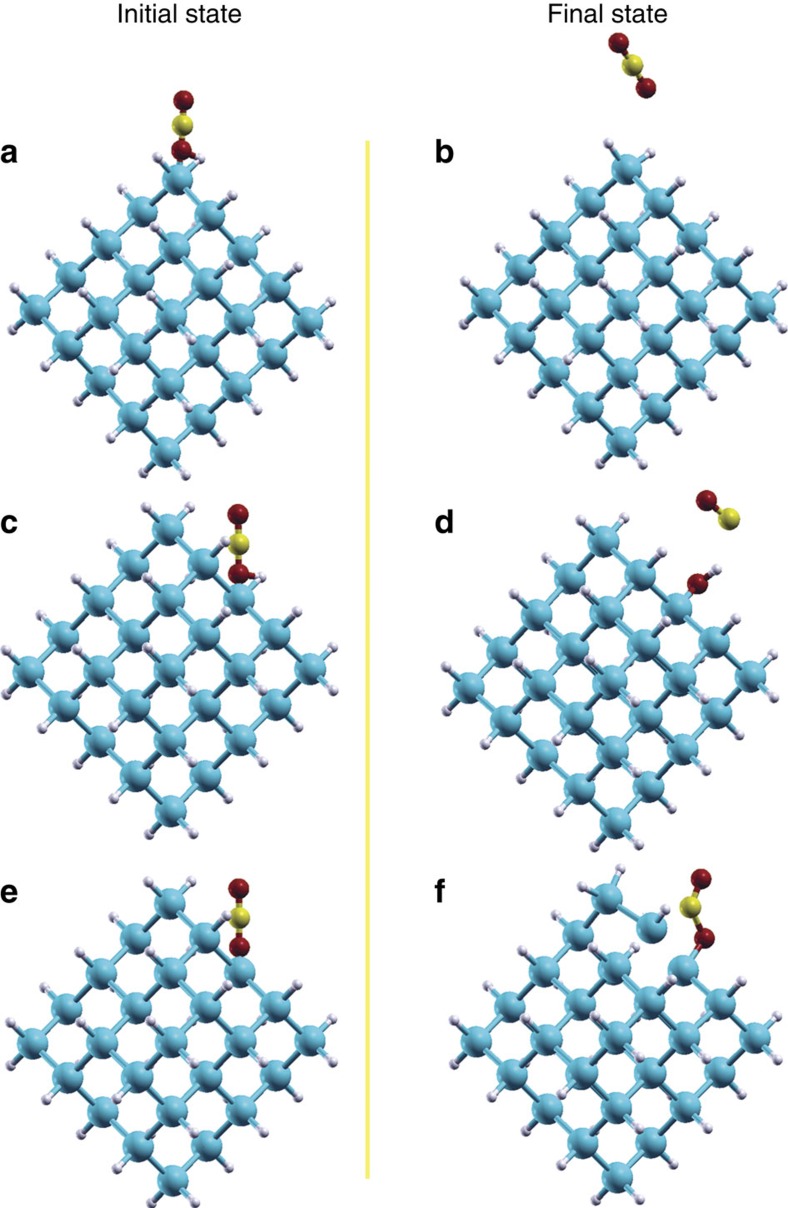
The DFT models. The initial models of CO_2_ adsorption on (**a**) SiH_2_, (**c**) SiH and (**e**) Si surface sites. The final optimized models of CO_2_ adsorption on (**b**) SiH_2_, (**d**) SiH and (**f**) Si surface sites.

While this paper reports an important first step in a brand new approach to CO_2_ reduction with still much to explore, we have conducted preliminary density functional theory (DFT) calculations to probe the identity, structural and chemical reactivity of the surface species involved in the reaction in more detail. We choose ∼1 nm Si_35_H_36_ model for this study. Our thermogravimetric analysis (TGA) confirms the H amount is comparable to the estimated amount from such models (Supplementary Fig. 7). The details of the computational method and models are given in the methods section. It is known that surface adsorption can lower the CO_2_ reduction potential in aqueous systems, making the reaction possible on semiconductors with a conduction band potential lower than the single-electron reduction potential of CO_2_ (refs 38, 39). It is conceivable that CO_2_ adsorption could similarly facilitate its reduction in gas phase reactions. Thus to explore the interaction between the gaseous CO_2_ molecule with the ncSi:H sample we also investigated computationally the CO_2_ adsorption on all possible surface sites. We placed the CO_2_ molecule in the vicinity of SiH_2_ (Si bonded with 2H's, Fig. 8a), SiH (Si bonded with 1H, Fig. 8c), and Si (Si with dangling bond Fig. 8e), surface sites and optimized each system. This analysis showed that the CO_2_ molecule will not interact with surface SiH_2_ and will remain intact (Fig. 8b). However, it will interact with the other two sites. At the dangling bond Si site CO_2_ molecularly adsorbs and bends as shown in Fig. 8f. The calculated binding energy of CO_2_ on the Si site is 0.63 eV. The C–O bond lengths for CO_2_ molecule increased from 1.16 to 1.22 Å and 1.53 Å, and the bond angle reduced from 180° to 121.2°. However the dangling bond Si site is not able to dissociate CO_2_. Interestingly, the SiH site managed to dissociate CO_2_ into CO and Si–OH endothermically (adsorption energy≈0.11 eV) making surface SiH the most favourable site for CO_2_ reduction reaction in ncSi:H system (Fig. 8d). The C–O and O–H bond lengths of the products are 1.17 and 1.01 Å, respectively, the Bader charges on O and C of CO are −1.79e and +1.74e, respectively, and Bader charge on O and H of OH are −2.07e and 0.00e, respectively. The strong bonds and charge distribution show that products formed by CO_2_ dissociation on the SiH site are very stable.

To examine if H_2_ can reinstate surface Si-H sites, the interaction of H_2_ with Si–OH formed after CO_2_ dissociation was also investigated. We simulated three different configurations. In the first configuration H_2_ was placed horizontally about 1.17 Å away from surface SiOH (Supplementary Fig. 10a), in the second configuration H_2_ was placed horizontally about 0.67 Å away from surface SiOH (Supplementary Fig. 10b) and the in third configuration H_2_ was placed vertically about 0.63 Å away from surface SiOH (Supplementary Fig. 10c). While optimizing all three configurations, H_2_ showed no interaction with the surface OH and remained intact moving away from the ncSi:H surface. This shows that surface hydroxides are very stable as also indicated by bond length and charge distribution discussed above and that H_2_ cannot easily recover the Si–H. This result is consistent with the literature^40^ and experimentally observed decrease in rate over time.

## Discussion

Unlike the reduction of CO_2_ by the molecular silanes, which goes through an intermediate species like SiOCH_2_OSi or SiOCHO^41^, in the case of ncSi:H we propose that the product CO directly leaves the surface of ncSi:H after O abstraction from CO_2_ without the C bonding to H. If our ncSi:H system went the same route as molecular silanes, we might expect to see CH_x_ surface species and ultimately CH_4_ and/or CH_3_OH as the products.

However we see no ^13^C labelled species on the Si surface in the IR spectrum, and no ^13^C labelled organic products other than CO. These diagnostics suggest that the reaction might not go through the insertion of CO_2_ into the Si-H bonds as found with molecular silanes with added catalysts. Instead, the insertion of O into the Si surface directs this heterogeneous reduction reaction to produce only CO. The Si–H surface of ncSi:H likely facilities the adsorption/binding of CO_2_ (ref. 42), and the large surface curvature and large surface to volume ratio likely facilitates the surface reactivity^43^, features which differentiate ncSi from bulk Si and molecular silanes. It is also important to note that the gas phase reaction temperature is quite different from that used with molecular silanes. Since the reaction also occurred in the dark, it is likely that the reduction reaction was able to be thermally driven without the need of exciton generation by light. Therefore, we attribute this unique gas-phase heterogeneous reduction reaction of CO_2_ by ncSi:H to its very small size, high surface area and highly reactive SiH surface. The impurity of F after HF etching during synthesis was negligible and should play a trivial role for very small Si nanocrystals with high surface curvature^44^, with only 1.43 at% F compared with 98.57 at% Si determined by XPS after heating at 60 °C in vacuum (spectra shown in Supplementary Fig. 11). With such vacuum thermal treatment, the sample was still active for CO_2_ reduction (Supplementary Fig. 5).

In the presence of H_2_, the production rate of CO from CO_2_ was enhanced. Apparently, the presence of H_2_ is beneficial for maintaining the SiH surface of ncSi:H in an un-oxidized state. Minimizing adventitious air oxidation especially in the light seems to be another way to preserve activity. Pertinently, light can be helpful in the reduction of CO_2_ via the oxidation of ncSi:H by the abstracted O from the CO_2_ itself. The potential of concentrated light to further enhance the CO_2_ reduction rate is also apparent. By optimizing the photothermal effect in ncSi:H it may prove possible to boost CO_2_ reduction rates to technologically significant values. If reduced to practice this would make earth abundant low cost silicon an attractive material as part of a global CO_2_ utilization strategy to meet IPCC emission targets by 2035.

Almost four decades have passed since the first report that sunlight can power the reduction of CO_2_ to carbon containing products in aqueous suspensions using semiconductor powder photocatalysts^45^. Since that time the photoreduction of CO_2_ by H_2_O or H_2_ have been studied in the aqueous and gas phase using almost every conceivable nanostructured composition imaginable^32^,^46^,^47^,^48^,^49^. While CO_2_ conversion rates and efficiencies of reported photocatalysts are still orders of magnitude below those required to inspire technological development^50^,^51^, progress towards achieving this objective has been steady and promising. One stumbling block along the road to success could prove to be the cost of scaling the photocatalyst to proportions of industrial relevance, because the compositions of the best photocatalysts are invariably comprised of rare and expensive elements. Overcoming this hurdle emphasizes the distinctiveness and significance of the work reported herein. It is indeed a surprising yet welcome discovery that plentiful, inexpensive and benign elemental silicon, synthesized in the form of ncSi:H made easily and cheaply from commercially available SiO, can exploit the reducing power of surface hydride to chemically reduce CO_2_ to CO—a synthon for making methanol or hydrocarbons by well-established methods—at scientifically impressive ∼mmol h^−1^ g_cat_^−1^ conversion rates. This advance bodes well for continued improvement by composition variations and doping methods, as well as size, shape and surface variations of ncSi:H, to achieve a higher rate and perhaps even transform the seemingly stoichiometric reaction to catalytic. The ultimate goal is to achieve technologically relevant CO_2_ utilization rates of mol h^−1^ g_cat_^−1^, which translates into Gt y^−1^ t_cat_^−1^.

Current global annual CO_2_ emissions from the use of fossil fuels amount to about 36 billion ton equivalents and are projected to reach 43 billion ton equivalents by 2030. In the war against climate change, the consensus is that carbon dioxide capture and storage alone cannot solve this problem and to have a meaningful effect needs to be combined with chemical and catalytic processes that convert the carbon dioxide into value-added chemicals and fuels^51^. It might well take something as simple and elegant as CO_2_ fixation by ncSi:H to solve such a monumental global problem.

## Methods

### Synthesis of ncSi in silicon oxide matrix

Solid SiO (purchased from Sigma-Aldrich, −325 mesh powder) was placed in a quartz reaction boat and transferred to a tube furnace. The samples were typically heated at a rate of 18 °C min^−1^ under a flow of 95% Ar/5% H_2_ to a peak processing temperature of 900 °C, then held at that temperature for 1 h before the furnace was allowed to cool to room temperature.

### Liberation of ncSi:H from silicon oxide matrix

For a typical batch synthesis, 0.3 g of 900 °C treated SiO powder was transferred to a Teflon beaker containing a mixture of 10 ml of 95% ethanol (aq. Sigma Aldrich) and 20 ml of 48% HF (aq. Sigma Aldrich). *Personnel should be well trained in the handling of HF*. The mixture was stirred for 2 h 50 min to fully etch away the silicon oxide matrix. The hydride-terminated ncSi were then extracted from the aqueous solution into pentane. The scale of the batch could be enlarged as long as the volume of the beakers and flasks are sufficient for safe handling of chemicals, for example, starting with 2 g of SiO powder instead of 0.3 g.

### Characterization

Powder X-ray diffraction was performed on a Bruker D2-Phaser X-ray diffractometer, using Cu Kα radiation at 30 kV. The nitrogen sorption experiments were performed at 77 K on a Quantachrome Autosob-1-C instrument. Prior to each adsorption measurement the samples were degassed at 60 °C overnight under vacuum. The specific surface area was determined using the Brunauer-Emmett-Teller (BET) equation, applied to the best linear fit within the range of 0.05≤*P*/*P*_0_≤0.35. The cumulative pore volume and pore size distribution were determined using non-local density functional theory (NL-DFT). FTIR was performed using a Perkin Elmer Spectrum-One FT-IR fitted with a universal attenuated total reflectance (ATR) sampling accessory with a diamond coated zinc selenide window. For the oxidation study of the ncSi:H on the KBr pellet, the FTIR spectra were acquired using the transmission mode without the ATR accessory. Diffuse reflectance of the samples was measured using a Lambda 1050 ultraviolet/VIS/NIR spectrometer from Perkin Elmer and an integrating sphere with a diameter of 150 mm. The ncSi:H aggregates morphology was characterized by SEM using a QUANTA FEG 250 ESEM. EPR measurements were performed at room temperature and 170 °C using a Bruker ECS-EMX X-band EPR spectrometer equipped with an ER4119HS cavity. An Oxford ITC503 temperature controller was utilized. Typical operating parameters were as follows: microwave frequency 9.363/9.393 GHz (for N_2_/CO_2_), microwave power 2.147/2.144 mW(for N_2_/CO_2_), modulation amplitude 1.000 G, sweep width 100 G centred at 3347.25 G, time constant 0.01 ms, total sweep time 300.00 s, number of scans 4. The EPR analysis was applied to dried ncSi:H samples, which were sealed in the 4 mm EPR tubes in the glove-box under a N_2_ gas atmosphere or sealed in CO_2_ gas. The TGA experiments were performed using a Discovery TGA (TA Instruments). The actual TGA test condition is described in Supplementary Fig. 7. X-ray photoelectron spectroscopy (XPS) was performed in an ultrahigh vacuum chamber with base pressure of 10^−9^ torr. The system used a Thermo Scientific K-Alpha XPS spectrometer, with an Al K_α_ X-ray source operating at 12 kV, 6 A and X-ray wavelengths of 1486.7 eV. The spectra were obtained with analyser pass energy of 50 eV with energy spacing of 0.1 eV. The sample for XPS analysis was prepared by drop casting ncSi:H in pentane on GaAs substrates and left in vacuum at 60 °C for several hours. The data analysis was carried out using Thermo Scientific Avantage software.

### Gas phase CO_2_ reduction measurements

Borosilicate glass microfiber filters were used as a substrate for gas phase reaction measurements to provide increased surface area as well as mechanical stability. A fresh dispersion of ncSi:H in pentane was dropped onto the filters and dried under N_2_ flow to yield films containing several mg of ncSi:H. Then the films were further dried under vacuum for at least 0.5 h, before placing into the reactor. A total pressure of ∼27 p.s.i. and a light intensity of one sun were the standard conditions for each run, otherwise the different conditions would be stated in the main text. The details of the reactor are listed as follows. These experiments were conducted in a custom fabricated 1.5-ml stainless steel batch reactor with a fused silica view port sealed with Viton O-rings. The reactors were evacuated using an Alcatel dry pump prior to being purged with the reactant gases H_2_ (99.9995%) and CO_2_ (99.999%) at a flow rate of 6 ml min^−1^ and a stoichiometry of 1:1 (stoichiometric for reverse water gas shift reaction). During purging, the reactors were sealed once they had been heated to the desired temperature. The reactor temperatures were controlled by an OMEGA CN616 6-Zone temperature controller combined with a thermocouple placed in contact with the sample. The pressure inside the reactor was monitored during the reaction using an Omega PX309 pressure transducer. Reactors were irradiated with a 1000 W Hortilux Blue metal halide bulb for a period of ∼22 h. For the test irradiated with concentrated light, the reactor with a volume of 11.8 ml was irradiated with a 300 W Xe lamp for a duration of 3 h. Product gases were analysed with a flame ionization detector and thermal conductivity detector installed in a SRI-8610 gas chromatograph with a 3′ Mole Sieve 13a and 6′ Haysep D column. Isotope tracing experiments were performed using ^13^CO_2_ (99.9 at% Sigma Aldrich). The reactors were evacuated prior to being injected with H_2_ followed by ^13^CO_2_. Isotope product gases were measured using an Agilent 7890 A gas chromatographic mass spectrometer (GC–MS) with a 60 m GS-CarbonPLOT column fed to the mass spectrometer.

### DFT simulations

DFT calculations were carried out using Quantum ESPRESSO^52^. The plane-wave-pseudopotential approach, together with the Becke-Lee-Yang-Parr^53^,^54^ exchange-correlation functional, and norm-conserving pseudopotentials was utilized throughout the analysis. All calculations are spin polarized. The kinetic energy cut-offs of 40 and 400 Ry were used for the smooth part of the electronic wave functions and augmented electron density, respectively. The self-consistent field convergence criterion was set to 1 × 10^−6^ Ry per Bohr and the structures were relaxed using a Davidson type diagonalization method until the magnitude of the residual Hellmann-Feynman force on each surface atom was less than 10^−3^ Ry per Bohr. The Brillouin zone integrations at gamma point were performed for full geometry optimization. To model ncSi:H we choose a spherical region of about 1 nm from the center of bulk diamond cubic silicon lattice and saturated the dangling bonds on the surface with hydrogen. The terminated Si atoms are classified into SiH and SiH_2_ types leading to a Si_35_H_36_ structure as illustrated in Supplementary Fig. 8. Blue and white spheres in Supplementary Fig. 8 represent the Si and H atoms, respectively. The cluster was located in a 30 × 30 × 30 Å cubic supercell. The relaxed structure is very similar to the previously reported structures^55^,^56^. To estimate the Si to H ratio in experimental samples that have mean size of about 3.5 nm, we also modeled ncSi:H of size 3.5 nm, as shown in Supplementary Fig. 9. The total number of atoms in this model is 1208 with 944 Si and 264 H atoms. However, since it is not feasible to work with such a large size model using DFT, we did not optimize this system and considered only the Si_35_H_36_ model for further DFT analysis.

### Data availability

All relevant data are available on request from the authors.

## Additional information

**How to cite this article**: Sun, W. *et al*. Heterogeneous reduction of carbon dioxide by hydride-terminated silicon nanocrystals. *Nat. Commun.* 7:12553 doi: 10.1038/ncomms12553 (2016).

## Supplementary Material

Supplementary Information Supplementary Figures 1-11 and Supplementary Tables 1-3.
