# Supplementary material for: Heterogeneous reduction of carbon dioxide by hydride-terminated silicon nanocrystals
Source: Nat Commun. 2016 Aug 23;7:12553. doi: 10.1038/ncomms12553 (PMC4996982; doi:10.1038/ncomms12553)
Supplement: Supplementary Information — Supplementary Figures 1-11 and Supplementary Tables 1-3. [file ncomms12553-s1.pdf]

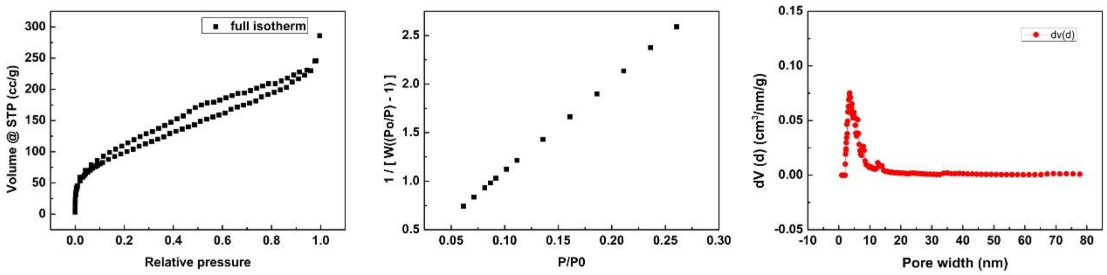

Supplementary Figure 1. Left: full isotherm of nitrogen adsorption and desorption displaying a type IV behavior with a hysteresis loop indicating disordered (textural) porosity. Middle: linear portion of $1/[W((P_0/P) - 1)]$ vs. relative pressure within the range of $0.5 \le P/P_0 \le 0.30$, used for the determination of BET surface area. Right: pore size distribution determined using NLDFT method – note that observed porosity is textural, due to packing of particles.

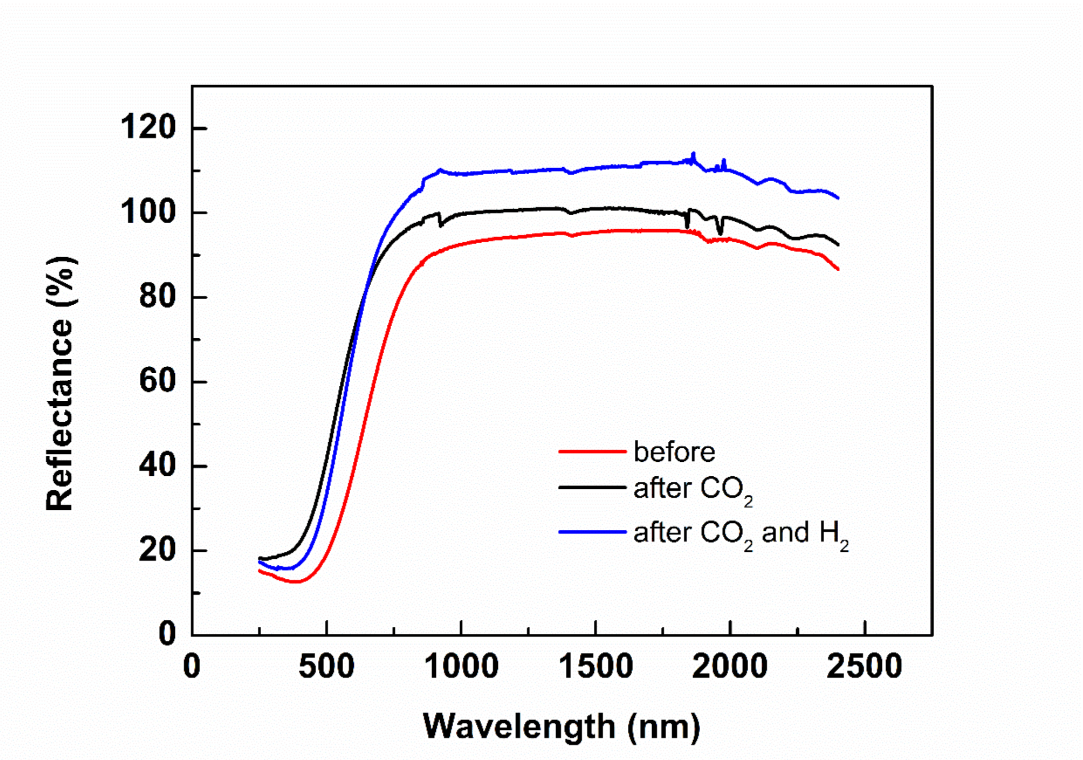

Supplementary Figure 2. UV-Vis diffuse reflectance spectra of the ncSi deposited on the glass fiber filter, before being put into the batch reactor, after reaction with pure CO_2, and after reaction with both CO_2 and H_2.

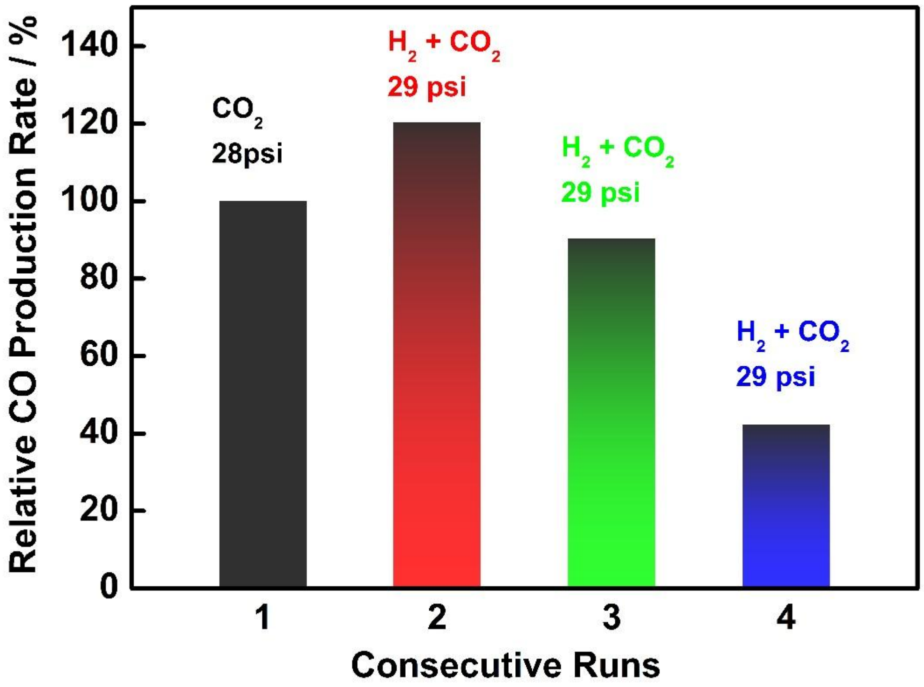

Supplementary Figure 3. Relative CO production rate was recovered when H_2 + CO_2 are both present in the second run. Each run was also over 20 h at 150 ℃. The rate for the first run with the presence of only CO_2 is defined as 100%, while the rates for following runs were normalized to it.

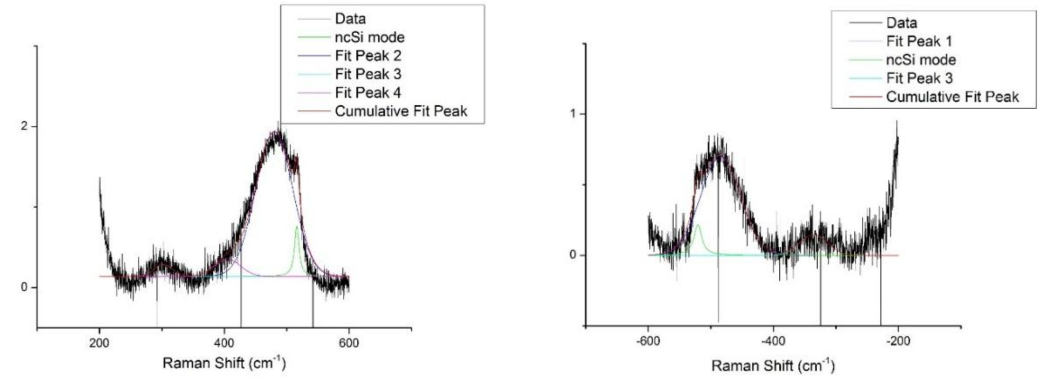

Supplementary Figure 4. Stokes and Anti-Stokes Raman spectra of the ncSi produced in SiO. The experimental details can be found in our previous work: *Nanoscale, 2016, 8, 3678-3684.* We employed the equation in *J. Phys. Chem. A 2008, 112, 1497-1501* and *Applied Physics Letters, 2001, 79, 937-939* for estimating the local temperature:

$$\frac{I_{\mathrm{ASt}}}{I_{\mathrm{St}}} = \left(\frac{\omega_{\mathrm{L}} + \omega_{\mathrm{V}}}{\omega_{\mathrm{L}} - \omega_{\mathrm{V}}}\right)^4 e^{-E_{\mathrm{R}}/(k\mathrm{T})}$$

Here $\mathrm{I_{St}}$ and $\mathrm{I_{ASt}}$ are the integral intensities of the Stokes (S) and anti-Stokes (AS) Raman scattering; T is the temperature in degrees Kelvin; E_R = $hc/\lambda_{excitation}$ − $hc/\lambda_{emission}$ = $hc\omega_{\mathrm{V}}$, where ω_{V} is the wave number of the Raman mode (cm^{-1}); ω_{L} is the wave number of the exciting laser light. After fitting of the peaks we obtain I_{ASt}/I_{St} = 0.5922 for the ncSi TO mode at 516.0 cm^{-1}. The estimated local T in this case is 876 K. The actual local T might be lower than the value calculated by this model due to various other physical factors such as heat diffusion, not considered in the model, nevertheless the photothermal heating effect induced by light has been well demonstrated.

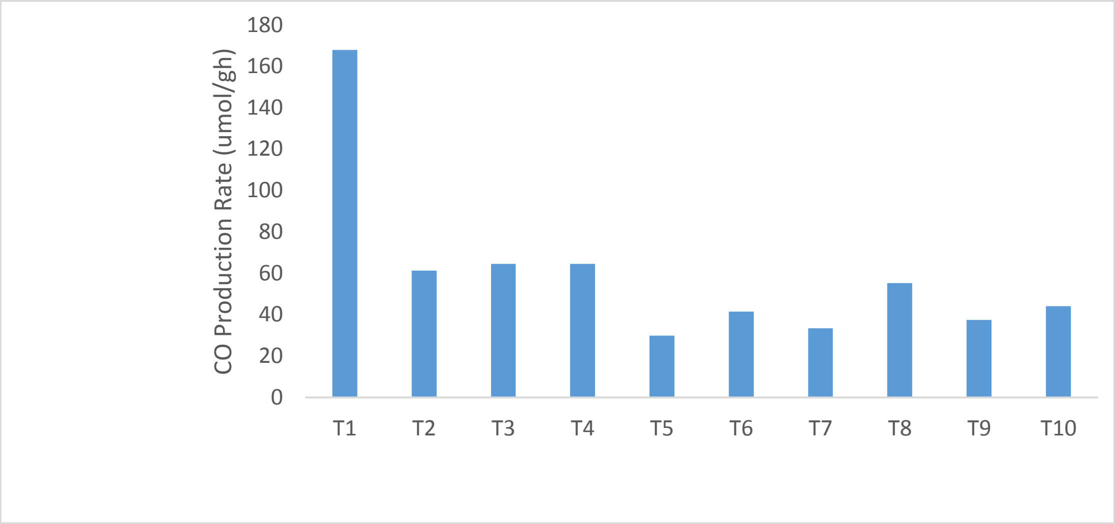

Supplementary Figure 5. The CO production rates of 10 cycling tests of a sample irradiated with more concentrated light (20 suns), without applying additional heating. The sample was heated at 60 °C overnight under vacuum in the reactor before the first test. Each test took ~3 h. The detailed design of this reactor used for high power irradiation will be published elsewhere.

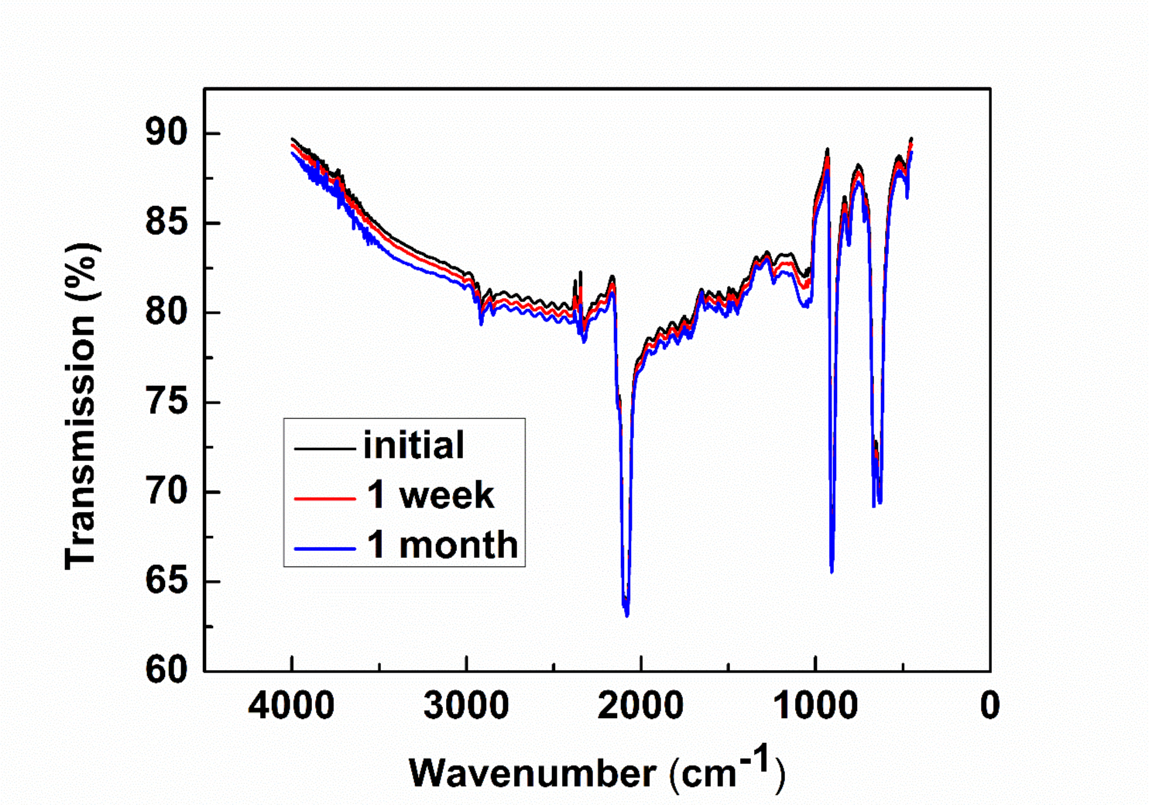

Supplementary Figure 6. IR spectra of ncSi:H drop-casted on a KBr plate over oxidation in a dark cabinet. The KBr plate was put in a desiccator open to the air with some drying agent below it. The peak at ~2100 cm^{-1} assigned to Si-H bond was barely decreased even over a month, while the Si-O-Si peak at ~1100 cm^{-1} was increased only slightly.

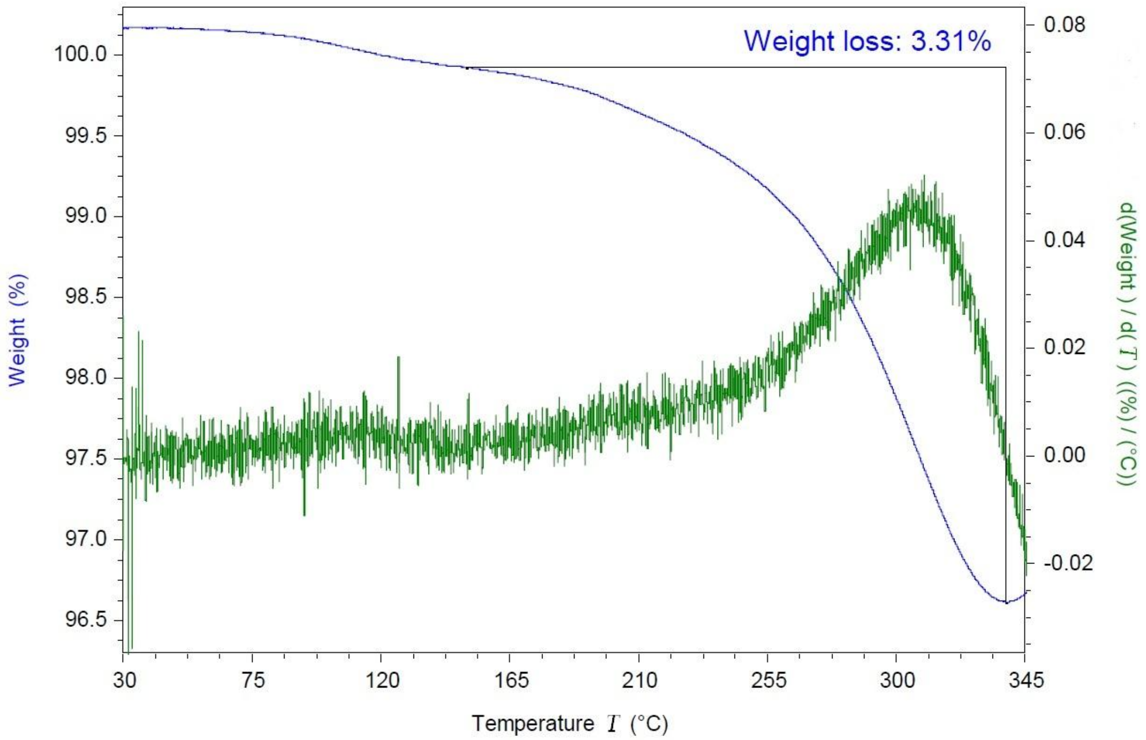

Supplementary Figure 7. Thermal gravimetric analysis (black) and differential thermal gravimetric analysis (green), which has been demonstrated to be an effective way to quantify the surface hydride on ncSi (***Chem. Mater***., 2010, **22**, 487–493). In this paper, the weight loss before 350 °C under Ar is mainly due to H loss accompanied by some loss of hydrocarbon, shown in Figure 11 in this reference. Inspired by this work, we also heated our freshly synthesized ncSi:H to 350 °C under He at a low ramp rate of 0.5 °C/min and with a high gas flow rate of 100 mL/min, and observed a 3.3% loss above 150 °C where the derivative starts to show significant increase of weight loss, corresponding to non-volatile species including H_2 loss. These observations provide an estimate that the surface Si-H accounts for up to ~3% of the total sample weight (this 3% weight loss may still contain a small amount of other non-volatile species), comparable to the models we constructed.

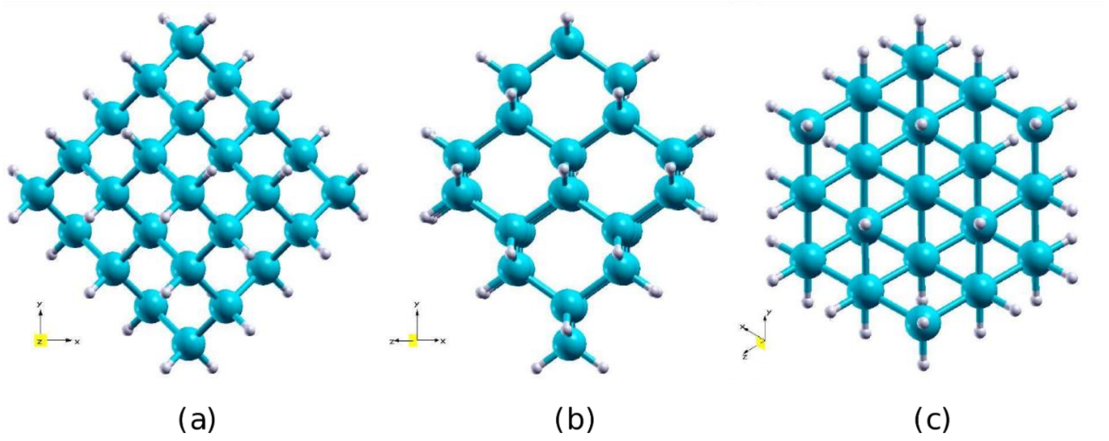

Supplementary Figure 8. Three projections of the atomic structure of a $Si_{35}H_{36}$ (~1 nm) nanocrystal. Blue and white spheres represent the Si and H atoms, respectively. The H weight percentage is about 3.4% estimated from this model.

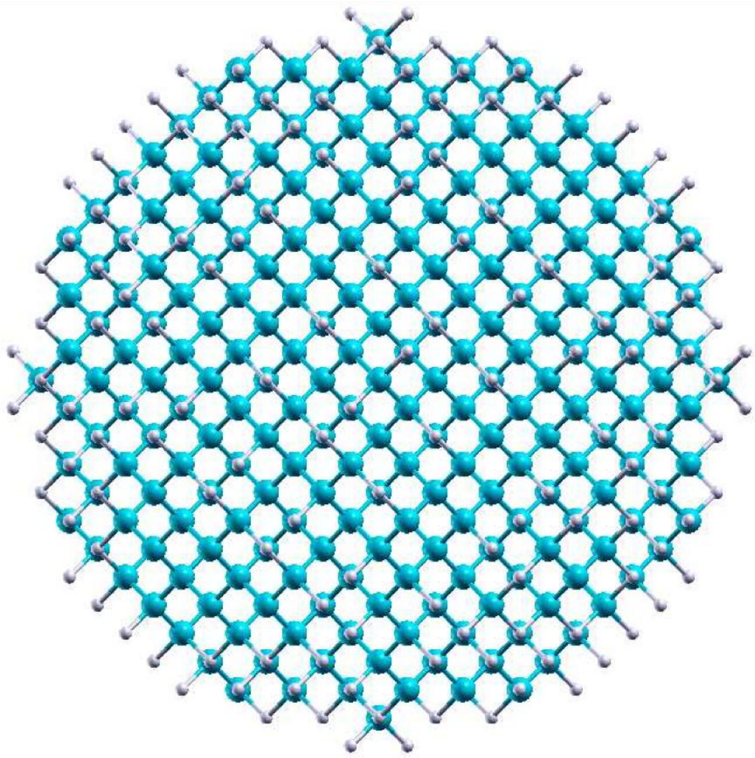

Supplementary Figure 9. The atomic structure of a Si:H nanocrystal of size ~3.5 nm. Blue and white spheres represent the Si and H atoms, respectively. This model contains 944 Si and 264 H atoms (Total number of atoms = 1208), with H weight percentage of about 1%. This analysis shows that the amount of surface H relative to the core Si will increase if the nanocrystal size decreases, but not significantly.

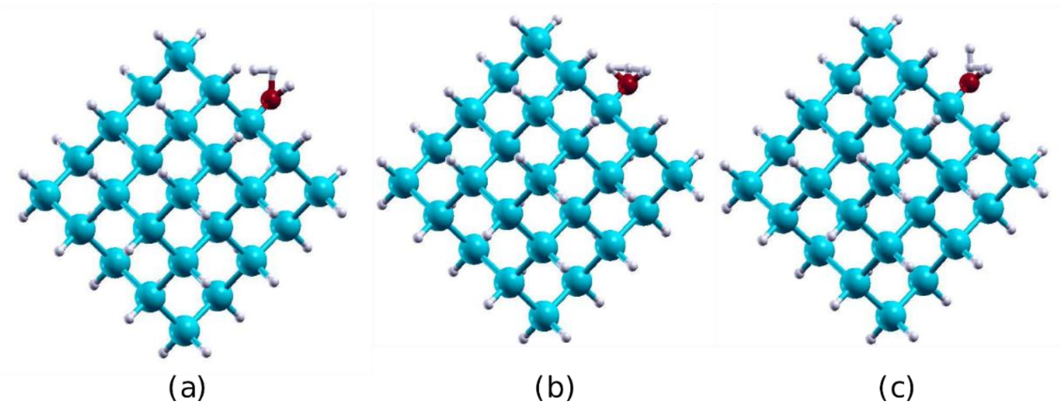

(a) (b) (c)

Supplementary Figure 10. The initial configurations considered for H_2 adsorption on ncSi:OH.

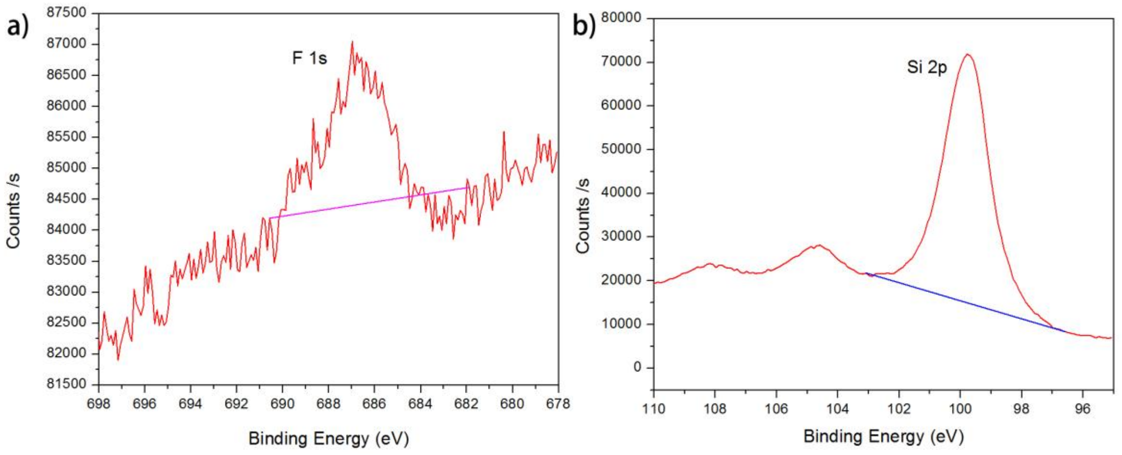

Supplementary Figure 11. F 1s (a) and Si 2p (b) core-level spectra of the sample. Background lines for atomic percentage analysis are also shown in the graphs, corresponding with the positions of fluoride (rather than Si-F) and Si(0) respectively, according to Nature Materials 9, 266–271 (2010).

Supplementary Table 1. ^{13}CO production rate of the CO_2 only and CO_2+H_2 tests compared to the total CO production rate (^{12}CO and ^{13}CO combined). The ratio of ^{12}CO to ^{13}CO produced were estimated by the ratio of the corresponding GCMS peak areas (mass-to-charge ratios of 28 and 29, respectively). The peaks were fitted assuming that they are Gaussian and areas were determined by the Peak Analyzer tool of OriginPro software. The ratio of ^{12}CO to ^{13}CO is then applied to the total CO production rates to obtain the corresponding production rates. The approach used in Table 1a to obtain the ^{12}CO:^{13}CO ratio is the same as that used to obtain the results shown in Supplementary Table 2, 3.

CO_2 only		CO_2+H_2		Large Reactor 20 Suns	
CO rate (nmol/g*h)	^{13}CO rate (nmol/gcat*h)	CO rate (nmol/g*h)	^{13}CO rate (nmol/gcat*h)	CO rate (µmol/g*h)	^{13}CO rate (µmol/gcat*h)
4569	3925	1967	1612	168	129
1184	792	1375	767	61	38
617	209	1119	408	65	48
480	92	881	133	64	30
444	56	775	75	30	10
420	41	702	50	42	20
365	31	638	35	33	5
303	23	594	28	55	4
		553	18	37	5
		455	24	44	6

Supplementary Table 2. ^{13}CO production rate compared to the total CO production rate (^{12}CO and ^{13}CO combined) in the sample stability tests.

sample storage time (days)		0	4	7	11	60
Stability Test	CO rate (μmol/g*h)	2.340	2.108	2.037	2.357	1.620
	^{13}CO rate (μmol/gcat*h)	1.272	1.636	1.463	1.104	0.962

Supplementary Table 3. ^{13}CO production rate compared to the total CO production rate (^{12}CO and ^{13}CO combined) in tests of the effect of temperature and light.

Temperature and Light Conditions	130 °C		150 °C		170 °C	
	Dark	Light	Dark	Light	Dark	Light
CO rate (µmol/g*h)	0.138	1.977	1.113	2.897	5.613	4.325
^{13}CO rate (µmol/gcat*h)	0.026	1.526	0.277	2.471	2.566	1.633
